# Role of health determinants in a measles outbreak in Ecuador: a case-control study with aggregated data

**DOI:** 10.1186/s12889-018-5163-9

**Published:** 2018-02-20

**Authors:** María F. Rivadeneira, Sérgio L. Bassanesi, Sandra C. Fuchs

**Affiliations:** 1 Programa de Pós-Graduação em Epidemiologia, Universidade Federal do Rio Grande do Sul, Porto Alegre, Brazil; 2 Instituto de Salud Pública, Facultad de Medicina, Pontificia Universidad Católica del Ecuador, Av. 12 de octubre 1076 y Roca, Quito, Ecuador; 3 Faculdade de Medicina, Universidade Federal do Rio Grande do Sul, Porto Alegre, Brazil; 4 Programa de Pós-Graduação em Epidemiologia, Universidade Federal do Rio Grande do Sul, Porto Alegre, Brazil

**Keywords:** Measles, Disease outbreaks, Case-control studies, Ecological studies

## Abstract

**Background:**

In 2011–2012, an outbreak of measles occurred in Ecuador. This study sought to ascertain which population characteristics were associated.

**Methods:**

Case-control study of aggregate data. The unit of analysis was the parish (smallest geographic division). The national communicable disease surveillance database was used to identify 52 case parishes (with at least one confirmed case of measles) and 972 control parishes (no cases of measles). A hierarchical model was used to determine the association of measles with population characteristics and access to health care.

**Results:**

Case parishes were mostly urban and had a higher proportion of children under 1 year of age, heads of household with higher educational attainment, larger indigenous population, lower rates of measles immunization, and lower rates of antenatal care visit attendance. On multivariate analysis, associations were found with educational attainment of head of household ≥8 years (OR: 0.29; 95%CI 0.15–0.57) and ≥1.4% indigenous population (OR: 3.29; 95%CI 1.63–6.68). Antenatal care visit attendance had a protective effect against measles (OR: 0.98; 95%CI 0.97–0.99). Measles vaccination was protective of the outbreak (OR: 0.97; 95%CI 0.95–0.98). The magnitude of these associations was modest, but represents the effect of single protective factors, capable of acting at the population level regardless of socioeconomic, biological, and environmental confounding factors.

**Conclusion:**

In Ecuador, the parishes with the highest percentage of indigenous populations and those with the lowest vaccination coverage were the most vulnerable during the measles outbreak.

## Background

Measles is a vaccine-preventable disease that is still associated with high morbidity and mortality rates. In 2015, despite of a 79% reduction in mortality from 2000 to 2015, 251,342 cases of measles were reported worldwide, with an estimated 134,200 deaths [1]. Even in countries where measles is controlled, the potential for outbreaks exists due to importations [2]. In addition, in countries with high overall vaccination coverage, measles outbreaks may originate in unvaccinated population clusters [3], such as groups facing social inequalities [4], poverty or an unfavorable family environment [5], ethnic [6] or religious minorities [7], floating populations [8], recent migrants, and those facing barriers to health care services [3] or vaccine hesitancy [7].

Ecuador has successfully controlled measles through regular vaccination and immunization campaigns. Ecuador introduced measles vaccination in the national calendar in 1974, administering a single dose. In 1999, a single dose of MMR vaccine was given in a campaign targeting children aged 12–23 months, which achieved 100% coverage. In 2009, a second dose of the vaccine was included into the regular schedule for 6 years-old children. Since 2016, there is a two-dose regimen for children 12 and 18 months old. In addition to regular vaccination, there have been mass vaccinations. Ecuador has carried out vaccination campaigns to broaden and strengthen the immunity of vulnerable populations, targeting different age groups. The campaigns carried out had national coverage, single doses of the vaccine were applied and the coverage was higher than 93% [9, 10]. Since 1998, national campaigns have been carried out every 4 years and coverage has exceeded 95%. In 2009, Ecuador adopted the Pan American Health Organization (PAHO) guideline for surveillance of suspected cases of measles, which requires clinical and laboratory confirmation of the diagnosis [11].

Between 1997 and 2010, only three cases of measles were reported in that country [12]. However, in 2011, a measles outbreak spread to nine out of the 24 provinces of Ecuador. By 2012, 329 cases had been confirmed; the highest incidence occurs in children under 1 year and 98% of the cases had no prior history of vaccination against measles [9]. We hypothesized that examining the characteristics of the population might provide an explanation for the outbreak and serve to prevent further outbreaks. Therefore, the aim of the present study was to identify population characteristics associated with the 2011–2012 measles outbreak in Ecuador through analysis of aggregate data.

## Methods

### Study design and population

An ecological case-control study was carried out to identify characteristics of the population that might have been associated with the 2011–2012 outbreak of measles in Ecuador. The analysis was based on cases with clinical and laboratory confirmation of measles. During the outbreak, patients with fever and maculopapular rash were considered as suspected measles cases. These patients underwent a laboratory workup consisting of IgM antibody testing in a blood sample obtained within the first 30 days after rash onset and measles PCR in a nasopharyngeal swab or urine sample obtained within the first 4 days after rash onset. In addition, patients with suspected measles who had been in direct contact with a laboratory-confirmed case within 21 days of symptom onset and lived in a parish affected by the outbreak were considered to have measles [13]. A governmental joint committee and members of PAHO validated all diagnoses of measles.

Health care, census, and measles outbreak data were aggregated at the level of the parish, the smallest geographic political division in Ecuador, which was defined as the unit of analysis. Parishes with at least one confirmed case of measles during the outbreak were classified as case parishes. Parishes with no recorded cases of measles during the outbreak were classified as control parishes. An epidemiological surveillance database/report of communicable diseases by the Ecuador Ministry of Public Health [14] was used to identify case and control parishes. The identification of parish case and parish control was based on epidemiological surveillance. The standard was ensured by the rate of suspected cases discarded (rate higher than 2 cases discarded per 100,000 inhabitants). Prior to the outbreak of measles, disease surveillance has been strengthened throughout the country though a certification process in order to eliminate measles, rubella and congenital rubella.

### Hierarchical analysis model

**Fig. 1:**
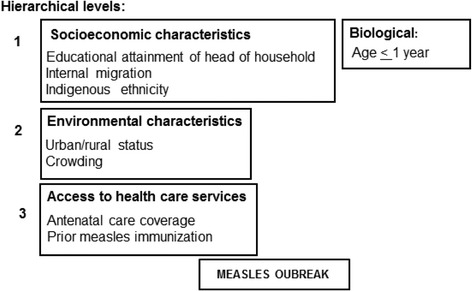
Determinants of health care associated with measles infection at parish level

A hierarchical model was developed to define independent variables that might be associated with the outbreak (Fig. 1). This modeling method has been previously described [15]. Briefly, it identifies characteristics that could increase the risk or protection against the outcome (parishes with measles) in chronological order, from the distal to the most proximal variables. The objective is a model with a sufficient number of variables to allow adequate data interpretation.

In the present study, a literature review was initially performed to identify biological, socioeconomic, and environmental characteristics, as well as variables relating to access to health care, associated with development of a measles outbreak. We also identified available databases for retrieval of existing data in Ecuador. Figure 1 shows the selected variables distributed in three hierarchical levels. The first cluster (distal hierarchical level) encompasses a cluster including socioeconomic variables that may increase the risk of infection and a cluster including a single biological variable (age ≤ 1 year) that reflects vulnerability to measles [6, 8, 16]. The second cluster (intermediate hierarchical level) encompassed environmental variables [16, 17], and the third cluster (the most proximal level to the measles outbreak) encompassed variables related to access to health care services [18, 19]. In all clusters, the selection of confounding factors was based on a *P* value < 0.10. Those which remained significantly associated with the case parishes (*P* <  0.10) entered into the model as confounding factors. In the multivariate analysis, associations with a P value < 0.05 were considered statistically significant.

Socioeconomic status was considered a distal determinant because its effects are reflected by other more proximal variables, such as environmental characteristics and access to health care services. Socioeconomic status has been reported to determine the access to measles vaccination [6, 16]. The only biological variable considered is a distal determinant because other characteristics do not influence it. Indigenous ethnicity was not considered a biological variable, but rather a marker of socioeconomic status [17]. Therefore, it was included in the socioeconomic cluster. Likewise, internal migration was included in that cluster, as it is strongly related to socioeconomic aspects. This decision was based on reports of measles outbreaks in Europe and Asia, which have been associated with floating populations of ethnic minorities with limited access to health care [8]. In the intermediate hierarchical level, environmental conditions are impacted by socioeconomic factors and may be a determinant of access to health care services [18], our most proximal hierarchical level. Access to health care services was represented in this study by surrogate variables, such as antenatal care coverage and measles immunization coverage, which are determined by socioeconomic, biological, and environmental factors [19, 20].

### Study variables

Parish with at least one confirmed case of measles was defined as the dependent variable.

Demographic, socioeconomic, and environmental variables for each parish were extracted from the Ecuador population census of 2010 [21]. The variables were categorized based on cut-off points, defined according to their distribution in the sample, to have comparable groups in relation to the dependent variable. The following variables were analyzed:

- Proportion of children under 1 year of age in the parish, categorized by the median into: < 3% of the population or ≥3% of the population.
- Average educational attainment of the head of household, categorized by the median into: < 8 years or ≥8 years.
- Internal migration was defined by the report of having moved the place of residence in the Ecuadorian territory to the parish. This information was collected in the 2010 national census, where people were asked: "Where were you living five years ago?” The number of people who have migrated to the parish was categorized into quartiles into: < 81, 81–198, 199–587, or > 588.
- Proportion of the parish population self-identified as indigenous, categorized by the median into: < 1.4% or ≥1.4%.
- Setting (urban or rural).
- Crowding expressed as percentage of households in the parish with more than three residents per bedroom, categorized in quartiles into: ≤14%; > 14 to < 21%; 21 to < 27%; or ≥27%.

Regarding access to health care, measles immunization was analyzed in terms of the percentage of individuals aged 6 months to 14 years who had received at least one dose of measles vaccine at any time up to data collection, in relation to the total parish population in this age range. These data were obtained from a survey on vaccination coverage conducted in 2011, at the beginning of the outbreak [9].

Antenatal care visits were analyzed in terms of the percentage of pregnant women with at least one visit to a public network health care center during pregnancy in relation to the overall number of live births in the parish. Health care reports [22] obtained from the Ecuador Ministry of Public Health provided information on access to antenatal care coverage.

### Data analysis plan

Independent variables (biological, socioeconomic, environmental, access to health care services) were expressed as absolute and relative frequencies. Because the dependent variable was dichotomous, logistic regression analysis was performed to determine independent variables statistically associated with the dependent variable. A *P* value < 0.10 was considered statistically significant. Subsequently, a multivariate analysis was conducted to assess the independent association between the outcome and biological, socioeconomic, environmental variables, and access to health care services. The odds ratios (OR) were calculated with 95% confidence intervals (95%CI). In this multivariate analysis, the hierarchical model [15] was used to assign variables within each level (Fig. 1). Therefore, the biological and socioeconomic variables were the first cluster that was included in the model, followed by environmental variables and, finally, by variables related to access to health care. Variables of the first cluster that remained significantly associated with the outcome were included in the multivariate analysis of the environmental cluster, and so on. Variables selected as confounding factors were socioeconomic determinants: educational attainment of head of household and internal migration; biological determinant: children under 1 year of age; environmental determinant: percent indigenous population; health care assessment: proportion of prior measles immunization and proportion of pregnant women attending antenatal care visit. In the model adjusted for confounding factors, associations with a *P* value < 0.05 were considered statistically significant. All analyses were carried out with the SPSS 21 software.

## Results

**Fig. 2:**
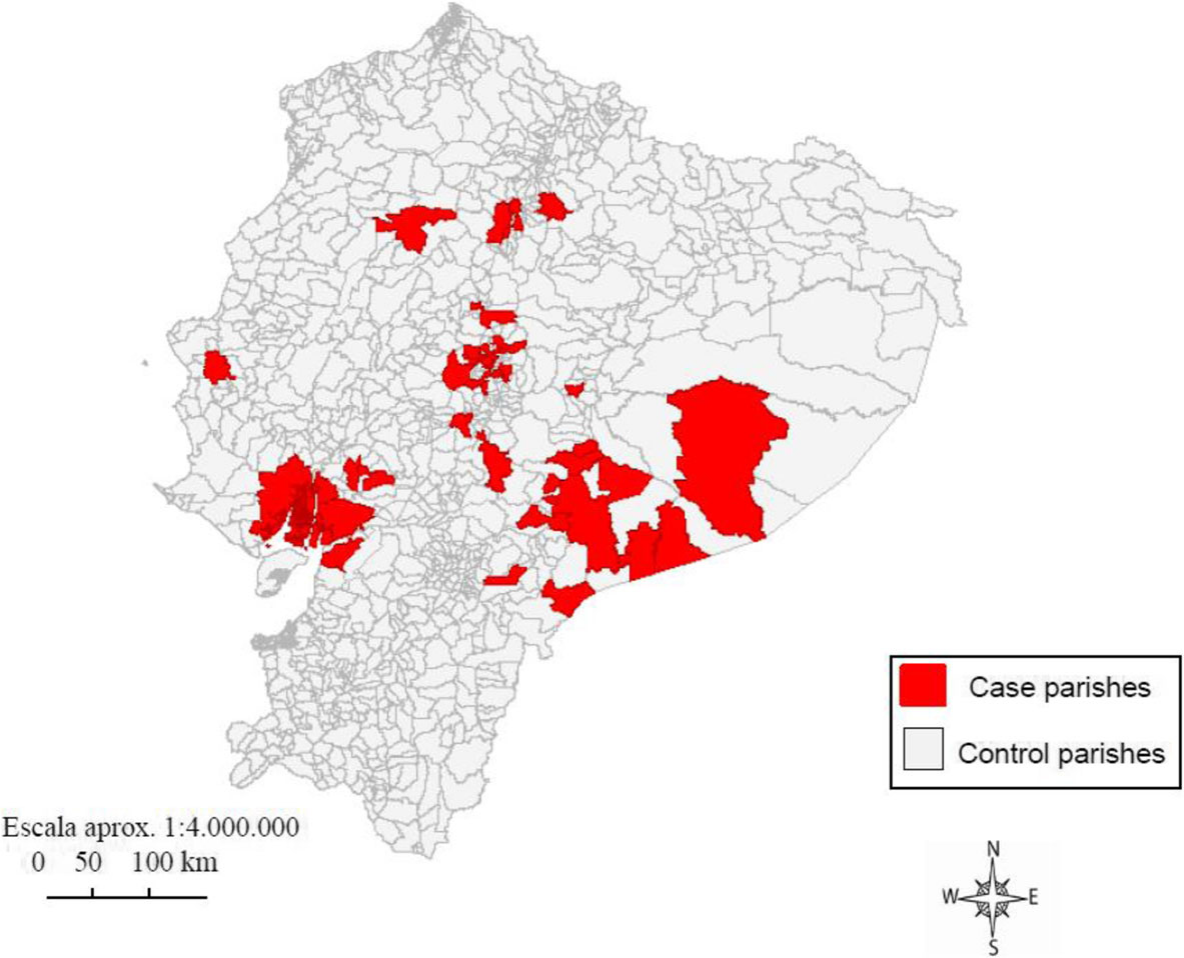
Map of case parishes during the 2011–2012 Ecuador measles outbreak

Among the 1024 parishes of Ecuador, 52 case parishes and 972 control parishes were identified. Figure 2 shows the geographic distribution of case parishes.

Table 1 shows that case parishes had 86.5% of children under 1 year of age versus 94.4% of control parishes. Higher proportion of heads of family with 8 or more years of education was observed among cases than control parishes. Approximately 44% of case-parishes were located in urban areas (vs. 20% of controls), had larger absolute number of internal migrants, indigenous population, but lower crowding rate. In addition, case parishes had lower mean rates of vaccination coverage and antenatal visits.

**Table 1:** Biological, socioeconomic, and environmental characteristics, and access to health care services in case and control parishes in Ecuador[n (%) or mean ± SD]

Characteristics	Case-Parishes	Control- Parishes	*P* value
Biological			
Children under 1 year of age (% parish population)			
< 3	45 (86.5)	918 (94.4)	0.019
≥ 3	7 (13.5)	54 (5.6)	
Socioeconomic			
Educational attainment of head of household (years)			
≥ 8	28 (53.8)	166 (17.1)	
< 8	24 (46.2)	806 (82.9)	< 0.001
Quartiles of the number of internal migrants			
< 81	7 (13.5)	250 (25.7)	0.001
81–198	8 (15.4)	247 (25.4)	
199–587	12 (23.1)	245 (25.2)	
≥ 588	25 (48.0)	230 (23.7)	
Percent indigenous population			
< 1.4	11 (21.2)	501 (51.5)	< 0.001
≥ 1.4	41 (78.8)	471 (48.5)	
Environmental			
Parish setting			
Rural	29 (55.8)	774 (79.6)	< 0.001
Urban	23 (44.2)	198 (20.4)	
Crowding (%)			
< 14	26 (50.0)	250 (25.7)	0.002
14–20	8 (15.4)	261 (26.9)	
21–26	7 (13.5)	219 (22.5)	
≥ 27	11 (21.1)	242 (24.9)	
Access to health care services			
Rate of prior measles immunization	79.90 ± 18.20	88.06 ± 12.06	0.02
Rate of pregnant women attending antenatal care visit^a^	83.90 ± 27.73	94.38 ± 41.14	< 0.001

^a^Percentage of pregnant women attending at least one pre-natal care visit were calculated using as the denominator the number of life births in the parish

Quantitative variables are shown categorized. Median was used to categorize: Children under 1 year of age, Educational attainment of head of household and Percent indigenous population. Quartiles were used for: Number of internal migrants and Crowding

Table 2 describes the non- and adjusted odds ratios for the associations of biological, socioeconomic, and environmental characteristics with measles. The association between the percentage of children under 1 year and case-control parishes showed an association, but it was only marginally significant after adjustment for socioeconomic characteristics. A positive association was observed between measles and proportion of heads of household with ≥8 years of education. Case parishes had significantly higher odds of having received migrants, but this association exhibited only a trend toward significance after adjustment for confounding factors. The odds ratio of measles cases was fourfold higher in parishes with > 1.4% indigenous population vs. parishes with < 1.4% indigenous population. This association remained significant even after adjustment for other variables. Regarding environmental characteristics, urban vs. rural setting was significantly associated with measles on univariate analysis. However, this association ceased to be significant when other variables were included in the model. Likewise, crowding did not hold a significant association after control for confounding factors (Table 2).

**Table 2:** Association of biological, socioeconomic, and environmental characteristics with measles in case parishes during the 2011–2012 measles outbreak in Ecuador

	OR (95%CI)^a^	OR (95%CI)^b^
Biological		
Children under 1 year of age (% population)		
< 3	1.00	1.00^b^
≥ 3	2.64 (1.14–6.14)	2.50 (0.99–6.34)
*P* value	0.024	0.053
Socioeconomic		
Head-of-household educational attainment (years)		
≥ 8	1.00	1.00 ^b^
< 8	0.18 (0.10–0.31)	0.29 (0.15–0.57)
P value	< 0.001	< 0.001
Quartiles of the number of internal migrants		
< 81	1.00	1.00^b^
81–198	1.16 (0.41–3.24)	1.07 (0.38–3.05)
199–587	1.75 (0.68–4.52)	1.44 (0.53–3.87)
≥ 588	3.88 (1.64–9.15)	2.56 (0.96–6.85)
*P* value	0.002	0.161
Environmental		
Percent indigenous population		
< 1.4	1.00	1.00^b^
≥ 1.4	3.97 (2.01–7.81)	3.29 (1.63–6.68)
P value	< 0.001	0.001
Parish setting		
Rural	1.00	1.00^c^
Urban	3.10 (1.76–5.48)	1.77 (0.84–3.76)
*P* value	< 0.001	0.135
Crowding (%)		
< 14	1.00	1.00^d^
14–20	0.29 (0.13–0.66)	0.34 (0.15–0.80)
21–26	0.31 (0.13–0.72)	0.49 (0.19–1.21)
≥ 27	0.44 (0.21–0.90)	0.46 (0.17–1.24)
*P* value	0.003	0.060

^a^Non-adjusted odds ratio (OR) and 95% confidence interval (95%CI)

^b^Odds ratio adjusted for children under 1 year of age, educational attainment of head of household, internal migration, and % indigenous population

^c^Odds ratio adjusted for the variables listed in ^b^ plus crowding

^d^Odds ratio adjusted for the variables listed in ^b^ plus parish setting

Please see footnote of Table 1 for variables definitions

Higher rates of prior measles vaccination were significantly associated with control-parish status on univariate analysis and also after adjustment for confounding factors (Table 3). Higher percentages of pregnant women who attended their antenatal care visits were also significantly associated with control-parish status. By comparing the parishes who presented outbreak in 2011 and 2012, it was found that characteristics of the population were similarly distributed in both years.

**Table 3:** Association between access to health care services with measles in case parishes during the 2011–2012 measles outbreak in Ecuador

	Crude OR (95%CI)^a^	Adjusted OR (95%CI)^b^
Rate (%) of prior measles immunization	0.97 (0.95–0.98)	0.97 (0.95–0.99)
P value	< 0.001	0.001
Rate (%) of pregnant women attending antenatal care visit	0.98 (0.97–0.99)	0.98 (0.97–0.99)
*P* value	0.002	0.002

^a^Crude odds ratio (OR)

^b^OR adjusted for the variables in Table 2

## Discussion

In this case-control study of aggregate data, we identified population characteristics that were associated with measles at the parish level during a measles outbreak in Ecuador. Among socioeconomic variables, associations were found with higher educational attainment of the head of household and higher proportion of indigenous population. Among environmental characteristics, crowding was inversely associated with measles. Both surrogate markers of access to health care services (prior measles vaccination and antenatal care visits) were also inversely associated with measles.

A noticeable finding of the present study was the significant association with parishes-cases where ≥1.4% of the population was indigenous. This result agrees with a previous study comparing the general population to Native Americans in the United States, which found that Native Americans were more susceptible to vaccine-preventable diseases because they faced precarious socioeconomic conditions and limited access to health care [20]. Along that line, a higher rate of indigenous populations was affected by the 1989–91 U.S. measles outbreak as compared to the white population [6]. Differences in measles immunization coverage, as estimated for children under 5 years of age, were suggested as a potential explanation for this increased incidence [6]. Thus, the independent association between measles and having ≥1.4% of the parish population identified as indigenous might have resulted from a gap in vaccination coverage rather than from socioeconomic characteristics. It is known that measles outbreaks may arise despite a high overall vaccination rate, for example in clusters of unvaccinated children [23, 24] or through importations [25].

The rate of antenatal care coverage applied here as an indicator of access to health care services, showed an inverse association with parishes-cases. Although the magnitude of the association of antenatal care visits with measles outbreak was modest, in practice, lack of antenatal care accounted for a 2% increased risk at the population level, regardless of confounding factors, which is not irrelevant. Our result confirmed the findings of a previous study, which shows that antenatal care was associated with fully immunized status among children in a district of Kabul, Afghanistan [26]; as well as another study in Pakistan, where lack of prenatal care was associated with incomplete vaccination [27]. However, this study performed an individual level analysis, while in our study it was a population level analysis. In any case, increase antenatal care coverage may be a strategy to prevent measles outbreak at a population level.

In this study, prior measles vaccination coverage showed an inverse association with the outbreak, suggesting a protective effect. That association appear trivial in magnitude, but represents the effect of the vaccination program based on weighted average of parishes. The result highlight that high vaccination coverage against measles (greater than 95%) is necessary to prevent outbreaks, especially among immunized populations [28, 29]. Otherwise, the effect of measles vaccination may be limited to generate herd immunity [30].

Unlike previously reported for other countries [19], we did not observe an association between the measles and internal migration in Ecuador. The loss of statistical significance on multivariate analysis suggests that internal migration was motivated by socioeconomic aspects, which limit access to vaccination [31]. Measles transmission through personal contact, via respiratory droplets [32], has been well documented and is facilitated by enclosed spaces [24] and overcrowding [33]. However, the actual development of measles depends on the presence both susceptible and infected individuals. In the present study, the inverse association between overcrowding and case-control parishes could actually suggest that it was the immune status of the contacts, not overcrowding, which was related to the outbreak. The direct association between educational level of the head of household and occurrence of measles appears to run counter to the protective effect of education on health outcomes. But, measles outbreaks have been reported to occur among intentionally unvaccinated individuals, particularly in areas of higher socioeconomic status [23]. In this study of aggregate data, one possible explanation for this finding is that case detection may have been more efficient in the parishes where heads of household have higher educational level, which would characterize reverse causality bias. Nevertheless, this finding could also indicate that there are other population characteristics related to the measles outbreak that were not investigated.

The use of a case-control design to evaluate characteristics of the population potentially associated with an outbreak of measles in a nationwide sample, constitutes a novel approach. However, some limitations should be considered when interpreting the results. One limitation is inherent to the use of aggregate data. The results of studies based on individuals are not directly comparable to those with data clustered at the population level. Therefore, findings cannot be extrapolated from population to individual level, since it would lead to an ecological fallacy or cross-level bias. In addition, data of antenatal care and measles vaccination coverage represent the respective averages for the parishes. These averages might have limited the magnitude of the association, since diluted the effect due to the inclusion of individuals who did not have prenatal care or vaccination. However, these associations are independent from others characteristics at population level and are consistent with previous evidence at individual level [17–19]. Another limitation is that further analysis are limited since the data were previously collected.

On the other side, facing measles outbreak decision-makers must take actions either to control or to prevent using strategies that work at the population level. Hence, identifying characteristics of population aggregates associated with measles outbreak may help to provide more efficient decisions. The data assessed in this study - biological, socioeconomic and environmental determinants - were based on information from a prior outbreak of measles, and were collected during the National Census in 2010. Data on measles vaccination and antenatal care came from information collected regularly by the country’s epidemiological surveillance systems. Even for vaccination coverage against measles, the data was collected initially at a time when there were no interventions at the national level. Therefore, we consider that the data analyzed in this study reflect the reality of population groups (parishes), and that there was no influence of the context of the outbreak on the data collected.

Strengths of this study should also be stressed, including that all measles cases were confirmed by clinical and laboratory assessment. Furthermore, reporting of measles cases to the national health surveillance system was supplemented by active case-finding, an approach that enabled collection of population data across the whole country. In addition, data entry and collection were carried out by one single agency, and case reports were controlled by the operational unit, improving the quality of information.

## Conclusions

In conclusion, the results of this study suggest that the outbreak of measles, in Ecuador, was associated with the proportion of indigenous population in the parish, reflecting the vulnerability of this group. In addition, the gap of coverage for measles vaccination (despite high overall coverage) seems an important determinant of the outbreak, as well the access to health care services. Further studies should investigate them among the strategies for prevention of measles outbreaks.

## Abbreviations

OR: Odds ratio
PAHO: Pan American Health Organization
